# Acid‐base disorders in sick goats and their association with mortality: A simplified strong ion difference approach

**DOI:** 10.1111/jvim.15956

**Published:** 2020-11-03

**Authors:** Diego E. Gomez, Sofia Bedford, Shannon Darby, Megan Palmisano, Robert J. MacKay, David L. Renaud

**Affiliations:** ^1^ Department of Large Animal Clinical Sciences College of Veterinary Medicine, University of Florida Gainesville Florida USA; ^2^ Department of Clinical Studies Ontario Veterinary College, University of Guelph Guelph Ontario Canada; ^3^ Department of Population Medicine Ontario Veterinary College, University of Guelph Guelph Ontario Canada

**Keywords:** unmeasured anions, hyperlactatemia, strong ion difference, clinical pathology, urolithiasis, haemonchus, pregnancy toxemia

## Abstract

**Objectives:**

To investigate the acid‐base status of sick goats using the simplified strong ion difference (*s*SID) approach, to establish the quantitative contribution of *s*SID variables to changes in blood pH and HCO_3_^−^ and to determine whether clinical, acid‐base, and biochemical variables on admission are associated with the mortality of sick goats.

**Animals:**

One hundred forty‐three sick goats.

**Methods:**

Retrospective study. Calculated *s*SID variables included SID using 6 electrolytes unmeasured strong ions (USI) and the total nonvolatile buffer ion concentration in plasma (A_tot_). The relationship between measured blood pH and HCO_3_^−^, and the *s*SID variables was examined using forward stepwise linear regression. Cox proportional hazard models were constructed to assess associations between potential predictor variables and mortality of goats during hospitalization.

**Results:**

Hypocapnia, hypokalemia, hyperchloremia, hyperlactatemia, and hyperproteinemia were common abnormalities identified in sick goats. Respiratory alkalosis, strong ion acidosis, and A_tot_ acidosis were acid‐base disorders frequently encountered in sick goats. In sick goats, the *s*SID variables explained 97% and 100% of the changes in blood pH and HCO_3_^−^, respectively. The results indicated that changes in the respiratory rate (<16 respirations per minute), USI, and pH at admission were associated with increased hazard of hospital mortality in sick goats.

**Conclusions and Clinical Importance:**

The *s*SID approach is a useful methodology to quantify acid‐base disorders in goats and to determine the mechanisms of their development. Clinicians should consider calculation of USI in sick goats as part of the battery of information required to establish prognosis.

**Abbreviations**
A^−^: total net negative charge of plasma proteins
A_tot_: total plasma concentration of nonvolatile weak acids
BHB: ß‐hydroxybutyrate
HCO_3_^−^: bicarbonate
H‐H: Henderson‐Hasselbalch approach
L‐lac^−^: L‐lactate
P_a_CO_2_: arterial partial carbon dioxide pressure
P_v_CO_2_: venous partial carbon dioxide pressure
PG: propylene glycol
SID: strong ion difference
SID_6_: strong ion difference measured using 6 electrolytes
SID_m_: measured strong ion difference
*s*SID: simplified strong ion difference approach
TPP: total plasma protein
USI: unmeasured strong ions

## 1 INTRODUCTION

Sick goats often are presented to tertiary hospitals with critical conditions such as acute diarrhea, pneumonia, gastrointestinal parasitism, pregnancy toxemia, mastitis, and urinary diseases such as urolithiasis. Regardless of presentation, these clinical abnormalities frequently result in marked acid‐base, fluid, electrolyte, and plasma protein disorders. The mechanisms contributing to acid‐base disorders in sick goats have not been extensively investigated. The few published studies focused on analysis of acid‐base disorders in goats with pregnancy toxemia^1^, ^2^ and urolithiasis^3^ using the traditional Henderson‐Hasselbach (H‐H) approach.^4^ In recent decades, different studies in sick horses,^5^, ^6^, ^7^, ^8^ and cattle^9^, ^10^, ^11^ have demonstrated the quantitative contribution of strong ions, plasma proteins, and PCO_2_ to the changes in plasma H^+^ activity and HCO_3_^−^ concentration by using the simplified strong ion difference (*s*SID) approach.^12^, ^13^, ^14^ The *s*SID proposes that 3 independent variables modify the plasma H^+^ activity. These 3 variables are the arterial partial carbon dioxide pressure (P_a_CO_2_), the strong ion difference (SID), and the total weak acid concentration (A_tot_^−^). The P_a_CO_2_ is regulated by the lungs and influences plasma H^+^ activity in a linear fashion.^13^, ^14^ Strong ion difference accounts for the contribution of the strong cations (Na^+^, K^+^, Ca^2+^, Mg^2+^) and strong anions (Cl^−^, L‐lactate^−^ [L‐lac^−^]), D‐lactate^−^, uremic acids, ketoacids, and unmeasured strong ions (USI) to the changes in plasma pH and HCO_3_^−^ concentration.^13^, ^14^ The A_tot_^−^ includes important nonvolatile weak buffers (mostly proteins and phosphate) that exert an independent effect on plasma H^+^ activity.^12^, ^13^, ^14^ The *s*SID approach has been used to determine the acid‐base balance of healthy goats.^15^ However, no published studies have evaluated acid‐base disorders in sick goats using the *s*SID.

Increased concentrations of USI other than L‐lac^−^ have been reported in critically ill human^16^, ^17^ and bovine patients.^9^, ^10^, ^11^ The source of these anions is unclear, but USI have been detected in human patients with sepsis,^18^, ^19^ decreased tissue perfusion,^20^ and kidney and liver injury.^21^, ^22^, ^23^ Increased concentrations of USI also were reported in calves with diarrhea, and the concentrations correlated with plasma concentrations of lipopolysaccharides.^24^ Higher concentrations of USI also were associated with increased risk of mortality in human^16^, ^25^ and bovine neonates.^26^ Currently, no information is available regarding the predictive value for mortality of admission concentrations of USI in sick goats.

Our objectives were to investigate the acid‐base status of sick goats using the *s*SID approach, to establish the quantitative contribution of the measured strong ion difference (SID_m_), PCO_2_, USI, and total plasma proteins (TPP) to changes of blood pH and HCO_3_^−^ concentration. Our study also aimed to investigate whether clinical, acid‐base, and biochemical variables, especially USI, on admission are associated with the mortality of sick goats. We hypothesized that *s*SID variables explain the majority of the changes in blood pH and HCO_3_^−^ concentration of sick goats, and that admission concentrations of USI and L‐lac^−^ are associated with mortality of sick goats.

## 2 MATERIALS AND METHODS

### 2.1 Study population and data collection

Medical records of all goats admitted at the Large Animal Hospital of the University of Florida, College of Veterinary Medicine, between January 2017 and September 2019 were reviewed. Goats were included in the study if they were >3 months of age, and if they had blood gas and TPP concentrations determined on admission before any treatment was instituted.

The following data were extracted from each record: age, breed, sex, heart rate (HR, beats per minute, bpm), respiratory rate (RR, respirations per minute, rpm), rectal temperature (T, °F), packed cell volume (PCV, %), TPP (g/dL), blood pH, venous partial pressure of carbon dioxide (P_v_CO_2_, mm Hg), bicarbonate concentration (HCO_3_^−^, mmol/L), electrolyte concentrations (Na^+^, K^+^, Mg^2+^, Ca^2+^, Cl^−^, L‐lac^−^, all in mmol/L), anion gap (AG, mEq/L), blood glucose concentration (mg/dL), blood ß‐hydroxybutyrate (BHB) concentration, and blood creatinine concentration (mg/dL). All blood gas analyses were corrected for the patient's rectal temperature using a standard method employed by the blood gas analyzer.

### 2.2 Sample collection and measurement techniques

Venous blood samples were collected in plastic blood collection tubes containing sodium heparin and obtained from the jugular vein at the time of admission. Venous blood gas analysis (VBGA), electrolyte, glucose, and creatinine concentrations were determined using a blood gas analyzer (Nova Biomedical Stat Profile, Novabio, Waltham, Massachusetts). The Na^+^, K^+^, Ca^2+^, Mg^2+^, and Cl^−^ concentrations were measured using ion‐selective electrode technology based on direct potentiometry. Blood pH and P_v_CO_2_ were measured using ion‐selective electrodes based on direct and indirect potentiometry, respectively, both based on the Nernst equation. The H‐H equation was used to calculated blood HCO_3_^−^ concentration (mmol/L): [HCO_3_^−^] = S × PCO_2_ × 10^(pH – pK1)^ with experimentally determined values for *S* (0.0307 [mmol/L]/mm Hg) and pK_1_ (6.095 at [NaCl] = 0.16 mmol/L) in plasma. The glucose, creatinine and L‐lac^−^ concentrations were determined using an enzymatic‐amperometric technique. The PCV was measured using a microhematocrit tube and TPP concentration was determined using refractometry (Cole‐Parmer, Vernon Hills, Illinois). The BHB concentration was also determined using an enzymatic‐amperometric technique (Abbott Diabetes Care, Alameda, California).

### 2.3 Calculations

The *s*SID variables were calculated using the formulas previously described for cattle^7^ as follows: strong ion difference (SID_m_, mEq/L) was calculated using the concentrations of 6 strong ions as: SID_6_ = (Na^+^ + K^+^ + Ca^2+^ + Mg^2+^) – (Cl^−^ + L‐lac^−^). The A_tot_ (mmol/L) was calculated as: A_tot_ = [3.43 × (TPP)], where TPP is in grams per deciliter. The USI (mEql/L) concentration was calculated as: USI = SID_6_ – HCO_3_^−^ – ([A_tot_^−^]/(1 + 10^(pKa‐pH)^)), where pK_a_ (7.08) is the effective dissociation constant of bovine plasma weak acids. Species‐specific values for K_a_ and A_tot_ have not been experimentally determined for goats, therefore values experimentally determined for calves^7^ were used.

### 2.4 Acid‐base disorders definition

Acid‐base disorders were diagnosed when the H‐H and *s*SID variables were outside of the reference ranges (Table 1).^7^, ^9^, ^26^

**TABLE 1:** Definition of acid‐base disorders using the Henderson‐Hasselbalch (H‐H) and the simplified strong ion difference (*s*SID) approach

Type of disorder	Approach	Variable	Acidosis	Alkalosis
Respiratory	H‐H and *s*SID	P_v_CO_2_ [34‐48 mm Hg]	↑ P_v_CO_2_	↓ P_v_CO_2_
Metabolic	H‐H	HCO_3_^−^ [20‐30 mmol/L]	↓ HCO_3_^−^	↑ HCO_3_^−^
		AG <24 mEq/L	↑ AG	N/A
	*s*SID	SID_6_ [38‐46 mEq/L]	↓ SID_6_	↑ SID_6_
		A_tot_ [19‐25 mmol/L]	↑ A_tot_	↓ A_tot_
		USI [−3 to 3 mEq/L]	↑USI	N/A

*Note*: Modified from References 10, 66.

Abbreviations: AG, anion gap; A_tot_ total plasma concentration of nonvolatile weak acids; HCO_3_^−^, bicarbonate; P_v_CO_2_, partial venous carbon dioxide pressure; SID, strong ion difference measured; USI unmeasured strong ions.

### 2.5 Statistical analysis

Descriptive analyses were performed on all variables. A Kolmogorov‐Smirnov test was used to assess normality of the data. Depending on the distribution, mean and standard deviation or median and range were calculated for each of the variables. Differences among goats with different clinical diagnoses were evaluated using the Steel‐Dwass test for nonparametric multiple comparisons.

The relationship between measured blood pH and HCO_3_^−^, and the *s*SID variables (SID_6_, USI, P_v_CO_2_, and A_tot_) was examined using a forward stepwise linear regression.^9^, ^10^, ^11^ The quantitative contribution of each variable was evaluated by order of entry into the model and change of the model *R* ^2^ value.^9^, ^10^, ^11^ The partial *R* or the potential contribution of the added variable for explaining the variation within the model was assessed by subtracting the R of the previous model from *R* of the current model.^9^, ^10^, ^11^ Residuals plots were examined to investigate the assumptions of the forward stepwise regression procedure. The regression results were confirmed using backward elimination.^9^ Evaluation of multicollinearity was carried out using the variance inflation factor of all significant predictors.^11^

To determine which clinicopathological variables were associated with the outcome of sick goats (discharged or euthanized or died), Cox proportional hazard models were constructed to assess the potential predictor variables and the outcome of interest. Days from admission to the hospital until discharged or died or euthanized was used as the time variable. Variables included into the analysis were sex, HR, RR, rectal T (T), creatinine, glucose, PCV, TPP, Na^+^, K^+^, Cl^−^, Ca^2+^, Mg^2+^, L‐lac^−^, pH, P_v_CO_2_, HCO_3_^−^, AG, SID_6_, and USI. The sex of the goats was categorized as male, female, or wether. Martingale residuals were plotted against the continuous variables to assess the linearity of each continuous predictor variable offered to the Cox proportional hazard models. If the variable failed to meet the linearity assumption, it was categorized as follows based on biologically relevant ranges^27^, ^28^: HR was categorized as <70, 70 to 80, and >80 bpm; RR as <16, 16 to 30, and >30 rpm; T as <101.3°F, 101.3°F to 103.5°F, and >103.5°F; blood creatinine concentration as ≤0.8 and >0.8 mg/dL; blood HCO_3_^−^ concentration as <20, 20 to 30, and >30 mmol/L; AG as ≤24 or >24 mmol/L; K^+^ as <4.2, 4.2 to 6, and >6 mmol/L; and Cl^−^ as <98 mmol/L, 98 to 110, and >110 mmol/L. Collinearity among variables was tested using Pearson correlation coefficients and if *r* > 0.6, only 1 variable was retained in the model. Variables with moderate statistical associations (*P* < .2) with the outcome of interest in univariable models subsequently were included in the multivariable analyses. A manual backward stepwise multivariable regression analysis was used for the different models. Variables were retained in the multivariable model if *P* < .05 or if the effect of removing the variable resulted in a change of at least 20% in the coefficient of a significant variable, indicating potential confounding. Two‐way interactions were evaluated between variables based on evidence from the literature and retained in the model if significant. Model fit was assessed using the test of proportional hazards for the Cox proportional hazards models. Harrell's *C* concordance statistic for each survival model was calculated to evaluate overall predictive ability. Harrell's *C* concordance statistic is calculated by taking all possible pairs of subjects consisting of 1 subject that experienced the event of interest and 1 subject that did not experience the event of interest. The statistic is the proportion of such pairs in which the subject that experienced the event had a higher predicted probability of experiencing the event than the subject that did not experience the event. A value of *C* = 0.5 corresponds to non‐informative prediction, whereas, *C* = 1.0 corresponds to perfect prediction.^29^ *P*‐values <.05 were considered significant.

For continuous variables that were significant in the final survival models, a cutoff was determined by receiver operator characteristics curve (ROC) analysis. The optimal cutoff point was based on the Youden's index and the corresponding sensitivity, specificity, and area under the curve were calculated for this cutoff point. Statistical analyses were performed using Minitab 19 (Minitab, LLC, State College, Pennsylvania) and STATA 14 (StataCorp LLC, College Station, Texas) statistical software.

## 3 RESULTS

### 3.1 Study population

One‐hundred forty‐three goats met the inclusion criteria. Seventy‐five (53%) goats were presented in 2017, 45 (31%) in 2018, and 23 (16%) in 2019. The age was recorded for 140 goats; median age on admission was 2 years (range, 3 months to 14 years). Of the 143 goats, 101 (71%) were females, 18 (12%) were males, and 24 (17%) were wethers. Breed distribution was similar to that of the general hospital population, with Nigerian Dwarf (n = 36, 25%) being the most commonly represented. Remaining breeds included La Mancha (n = 24, 17%), Nubian (n = 20, 14%), Oberhasli (n = 17, 12%), Boer (n = 8, 6%), Pygmy (n =7, 5%), Toggenburg (n = 5, 3%), Alpine (n = 2, 1%), and other breeds (n = 24, 17%). The values (mean, SD, median and range) for HR, RR, T, VBGA, electrolyte concentrations, TPP, and the H‐H and *s*SID variables for all 143 goats are presented in Supplementary Table 1.

### 3.2 Electrolyte, biochemical and acid‐base abnormalities in sick goats

Nineteen (13%) goats were diagnosed with acute diarrhea, 19 (13%) with urolithiasis, 16 (11%) were presented for dystocia, 14 (10%) with haemonchosis, 13 (9%) with pregnancy toxemia, 10 (7%) with pneumonia, and 7 (5%) with clinical mastitis. Forty‐five (32%) goats were diagnosed with other diseases including azalea toxicity (n = 4, 3%), hypocalcemia (n = 3, 2%), simple indigestion (n = 3, 2%), vagal indigestion (n = 3, 2%), neoplasia (n = 3, 2%), ruminal foreign body (n = 2, 1%), and peritonitis (n = 2, 1%).

The results for blood gas analysis, electrolyte concentrations, TPP, H‐H, and *s*SID variables for 98 goats with selected diseases are presented in Table 2. Goats suffering from haemonchosis had significantly lower PCV (median, 8%; range, 7%‐18%) and TPP (median, 4.4 g/dL; range, 3.5‐7.0 g/dL) than did goats from other groups (*P* < .05). Blood glucose concentration was higher (median, 193 mg/dL; range, 90‐325 mg/dL) in goats with diarrhea than in other groups (*P* < .05), except the urolithiasis group. Goats with urolithiasis had blood creatinine concentration (median, 3.4 mg/dL; range, 0.6‐12.5 mg/dL) higher than did the other groups (*P* < .05), except the clinical mastitis group, whereas goats with dystocia had higher concentration of Cl^−^ (median, 115 mmol/L; range, 108‐119 mmol/L) than did the other groups (*P* < .05). The A_tot_ was signifficantly lower in goats with haemonchosis (median, 15 mmol/L; range, 12‐23 mmol/L) than other groups (*P* < .05, for all comparisons). Goats with pregnancy toxemia had a higher concentration of USI (median, 3.8 mEq/L; range, −2.4 to 14 mEq/L) than did goats with acute diarrhea, urolithiasis, haemonchosis, and clinical mastitis (*P* < .05). Concentrations of BHB (mmol/L) were only measured in goats with pregnancy toxemia with a median of 3 mmol/L (range, 1.2‐8 mmol/L; Table 2).

**TABLE 2:** Admission results of blood gas, electrolytes, acid‐base and selected laboratory variables of 98 sick goats with selected diseases

Variable	Acute diarrhea	Pneumonia	Urolithiasis	Haemonchus infection	Pregnancy toxemia	Clinical mastitis	Dystocia
Cases	19	10	19	14	13	7	16
PCV (%)	37^be^ 29 to 55	30^bd^ 29 to 60	29^cd^ 19 to 47	8^a^ 7 to 18	25^cd^ 12 to 42	32^bcd^ 25 to 39	30^cd^ 23 to 52
TPP (g/dL)	7.1^b^ 6.4 to 8.2	6.5^bc^ 4.8 to 9.4	6.3^c^ 4 to 7.3	4.4^a^ 3.5 to 7	6.6^bc^ 5.5 to 7.5	6.5^bc^ 6.2 to 8	6.5^bc^ 5 to 8
Glucose (mg/dL)	193^a^ 90 to 325	113^bc^ 34 to 284	131^ac^ 67 to 380	105^bc^ 23 to 190	95^bc^ 51 to 160	62^bc^ 37 to 99	119^bc^ 67 to 284
Creatinine (mg/dL)	1.1^bc^ 0.4 to 3	0.9^bc^ 0.4 to 1.7	3.4^a^ 0.6 to 12	0.75^bc^ 0.5 to 1.2	1^bc^ 0.7 to 2	1^ac^ 0.6 to 3	1^bc^ 0.6 to 2.4
Na^+^ (mmol/L)	141^a^ 134 to 151	144^a^ 137 to 146	143^ab^ 131 to 152	138^a^ 135 to 148	143^ab^ 136 to 150	141^ab^ 135 to 147	145^b^ 141 to 152
K^+^ (mmol/L)	3.6^b^ 2.4 to 4.6	4^ab^ 2 to 5.2	4.6^a^ 2.2 to 10	3.5^b^ 2 to 4.6	3.8^b^ 2.4 to 4.5	4^ab^ 2.8 to 4.6	4.1^ab^ 2.8 to 4.6
Cl^−^ (mmol/L)	108^ad^ 96 to 112	113^abc^ 87 to 119	109^ab^ 100 to 119	111^bc^ 94 to 118	111^bc^ 108 to 118	112^bc^ 108 115	115^c^ 108 119
Ca^2+^ (mmol/L)	1.1^a^ 0.9 to 1.2	1.1^a^ 0.8 to 1.4	1.2^a^ 0.8 to 1.6	1.1^a^ 1 to 1.2	1.1^a^ 11 to 1.3	1.1^a^ 1 to 1.2	1.1^a^ 1 to 1.2
Mg^2+^ (mmol/L)	0.5^a^ 0.4 to 1	0.5^a^ 0.3 to 0.7	0.6^a^ 0.4 to 1.3	0.5^a^ 0.4 to 0.7	0.5^a^ 0.4 to 0.6	0.5^a^ 0.4 to 0.6	0.5^a^ 0.4 to 0.6
L‐lac^−^ (mmol/L)	3.8^a^ 0.8 to 7.2	2.1^bc^ 0.4 to 7.2	1.5^bc^ 0.5 to 8.6	2.8^ac^ 1 to 10	1.4^ac^ 0.3 to 7	2.1^ac^ 0.3 to 5.6	2.6^ac^ 0.4 to 7.2
pH	7.44^ab^ 7.30 to 7.50	7.46^ab^ 7.38 to 7.52	7.47^b^ 7.36 to 7.55	7.46^b^ 7.37 to 7.53	7.37^a^ 7.18 to 7.52	7.46^ab^ 7.38 to 7.48	7.46^8ab^ 7.39 to 7.51
P_v_CO_2_ (mm Hg)	31^a^ 22 to 42	26^ab^ 21 to 46	29^ab^ 20 to 60	26^ab^ 17 to 43	24^b^ 18 to 32	25^ab^ 16 to 30	25^b^ 21 to 31
HCO_3_^−^ (mmol/L)	20^bc^ 14 to 30	19^bd^ 14 to 34	21^bc^ 15 to 33	19^bd^ 11 to 33	16^ad^ 7.7 to 21	18^bd^ 11 to 22	19^bd^ 14 to 26
AG (mEq/L)	16^cd^ 13 to 22	16^abc^ 5.3 to 25	15^b^ 8 to 19	12^c^ 7 to 17	19^a^ 11 to 27	15^abc^ 12 to 19	17^abd^ 13 to 19
SID_6_ (mEq/L)	35^ab^ 30 to 44	35^ab^ 29 to 41	38^a^ 26 to 49	29^b^ 19 to 46	33^ab^ 28 to 40	30^ab^ 23 to 37	30^ab^ 23 to 21
A_tot_ (mmol/L)	24^b^ 22 to 28	22^bc^ 16 to 32	21^c^ 14 to 25	15^a^ 12 to 23	22^bc^ 29 to 26	22^bc^ 21 to 27	22^bc^ 17 to 28
USI (mEq/L)	−2.9^b^ −5 to 2.1	−0.8^ab^ −4.6 to 6.6	−1.4^b^ −3.5 to 3.3	−1.9^b^ −3.8 to 1.6	3.8^a^ −2.8 to 14	−3.5^b^ −5.1 to 1.6	−0.8^ab^ −3.3 to 6.6

*Notes*: Data presented as median and lower and higher range. Different letters within a row indicated a statistically significant difference (*P* < .05). *P*‐values were obtained using the Steel‐Dwass test for nonparametric multiple comparisons.

Abbreviations: AG, anion gap; A_tot_, total plasma concentration of nonvolatile weak acids; HCO_3_^−^, bicarbonate; L‐lac^−^, L‐lactate; PCV, packet cell volume; P_v_CO_2_, venous partial carbon dioxide pressure; SID, strong ion difference; TPP, total plasma proteins; USI, unmeasured strong ions.

The electrolyte, TPP, and acid‐base disorders detected in 143 sick goats are presented in Table 3. Overall, the most common electrolyte abnormalities in sick goats were hyper‐L‐lactatemia (n = 96, 67%), hypokalemia (n = 89, 62%), and hyperchloremia (n = 76, 53%). Hyponatremia was present in 6 animals (4%) and hypernatremia was not detected in any of the goats. Hypoproteinemia frequently was diagnosed in goats with haemonchosis (n = 11, 78%).

**TABLE 3:** Acid‐base, electrolyte, and total plasma protein disorders detected on admission in 143 sick goats with selected diseases

	Acute diarrhea	Pneumo	Urolith	Haemon	Preg. Tox.	Mastitis	Dystocia	Others
Cases (n)	19	10	19	14	13	7	16	45
Na^+^								
Hyponatremia	0 (0%)	1 (10%)	2 (11%)	0 (0%)	0 (0%)	0 (0%)	0 (0%)	3 (7%)
Cl^−^								
Hypochloremia	2 (11%)	2 (20%)	0 (0%)	1 (7%)	0 (0%)	0 (0%)	0 (0%)	0 (0%)
Hyperchloremia	2 (11%)	5 (50%)	8 (42%)	8 (57%)	7 (54%)	6 (86%)	15 (94%)	25 (56%)
K^+^								
Hypokalemia	15 (79%)	7 (70%)	4 (21%)	11 (79%)	10 (77%)	4 (57%)	9 (56%)	29 (64%)
Hyperkalemia	0 (0%)	0 (0%)	3 (16%)	0 (0%)	0 (0%)	0 (0%)	0 (0%)	0 (0%)
L‐Lactate^−^								
Hyperlactatemia	18 (95%)	4 (40%)	10 (53%)	13 (93%)	7 (54%)	5 (71%)	12 (75%)	27 (60%)
TPP								
Hypoprotein	0 (0%)	2 (20%)	6 (32%)	11 (79%)	5 (38%)	0 (0%)	6 (38%)	13 (29%)
Hyperprotein	1 (5%)	2 (20%)	0 (0%)	0 (0%)	0 (0%)	0 (0%)	1 (6%)	4 (9%)
pH								
Acidemia	0 (0%)	0 (0%)	0 (0%)	0 (0%)	2 (15%)	0 (0%)	0 (0%)	1 (2%)
Alkalemia	2 (11%)	2 (20%)	7 (37%)	2 (14%)	1 (8%)	0 (0%)	4 (25%)	10 (22%)
PvCO_2_								
Resp. acidosis	0 (0%)	0 (%)	1 (5%)	0 (0%)	0 (0%)	0 (0%)	0 (0%)	0 (0%)
Resp. alkalosis	16 (84%)	8 (80%)	14 (74%)	12 (86%)	13 (100%)	7 (100%)	16 (100%)	39 (87%)
HCO_3_^−^								
Met. acidosis	5 (26%)	6 (60%)	4 (21%)	8 (57%)	11 (85%)	6 (86%)	10 (63%)	18 (40%)
Met. alkalosis	0 (0%)	1 (10%)	3 (16%)	1 (7%)	0 (0%)	0 (0%)	0 (0%)	1 (2%)
AG								
Acidosis	0 (0%)	1 (10%)	0 (0%)	0 (0%)	2 (15%)	0 (0%)	0 (0%)	2 (4%)
SID_6_								
Acidosis	15 (79%)	9 (90%)	11 (58%)	13 (93%)	12 (92%)	7 (100%)	14 (88%)	36 (80%)
Alkalosis	0 (0%)	0 (0%)	0 (0%)	1 (7%)	0 (0%)	0 (0%)	0 (0%)	0 (0%)
A_tot_								
Acidosis	7 (37%)	4 (40%)	1 (5%)	0 (0%)	3 (23%)	3 (43%)	2 (13%)	12 (27%)
Alkalosis	0 (0%)	2 (20%)	2 (10%)	11 (71%)	2 (15%)	0 (0%)	4 (26%)	7 (15%)
USI								
Acidosis	0 (0%)	0 (0%)	1 (5%)	0 (%)	8 (62%)	0 (0%)	1 (6%)	4 (9%)

Abbreviations: AG, anion gap; A_tot_, total plasma concentration of nonvolatile weak acids; Haemon, haemonchus infection; HCO_3_^−^, bicarbonate; Hyperlactate, hyperlactatemia; Hyperprotein, hyperproteinemia; Hypoprotein, hyporproteinemia; Pneumo, pneumonia; PvCO_2_, venous partial carbon dioxide pressure; SID, strong ion difference; TPP, total plasma proteins; USI, unmeasured strong ions.

Based on the H‐H approach, the most common acid‐base disorders in sick goats were respiratory alkalosis (n = 125, 87%) and metabolic acidosis (n = 68, 48%). Anion gap acidosis was detected in only 2 (1%) goats. When using the *s*SID approach, the most common acid‐base disorders were respiratory alkalosis (n = 125, 87%) and SID_6_ acidosis (n = 117, 82%). A USI acidosis was diagnosed in 14 (10%) goats, whereas A_tot_ alkalosis was present in 17 (12%), and A_tot_ acidosis in 84 (59%) goats.

The most common *s*SID acid‐base disorders in goats with acute diarrhea, pneumonia, urolithiasis, mastitis, and dystocia were respiratory alkalosis and SID_6_ acidosis. In goats with pregnancy toxemia, respiratory alkalosis, SID_6_ acidosis and USI acidosis frequently were detected (n = 8, 62%). Hypoproteinemic (A_tot_) alkalosis and hyperproteinemic (A_tot_) acidosis were commonly detected in goats with haemonchosis (n = 11, 72%) and acute diarrhea (n = 7, 37%), respectively.

### 3.3 *s*SID variables and blood pH and HCO_3_^−^

The results of forward stepwise regression using venous blood pH and calculated HCO_3_^−^ concentrations as dependent variables showed that, in sick goats, P_v_CO_2_ was the most important variable associated with variations in the measured blood pH and calculated HCO_3_^−^. The SID_6_, USI, and A_tot_ also were associated with changes in the measured blood pH and HCO_3_^−^ (Table 4).

**TABLE 4:** Results of the stepwise linear regression of measured blood pH and calculated HCO_3_^−^ as dependent variables versus jugular venous values of the simplified strong ion difference variables of 143 sick goats

	Order of entry	Variable	Partial *R* ^2^	Model *R* ^2^	VIF
pH	1	SID_6_	9.5	9.5	2.46
	2	USI	13.5	23	1.60
	3	A_tot_	24	47	1.57
	4	P_v_CO_2_	50.3	97.3	1.94
					
HCO_3_^−^	1	P_v_CO_2_	59.8	59.8	2.6
	2	SID_6_	12.1	71.9	1.59
	3	A_tot_	8.4	80.3	1.55
	4	USI	19.6	99.9	2.09

*Note*: Modified from Reference 11.

Abbreviations: A_tot_ total plasma concentration of nonvolatile weak acids; P_v_CO_2_ partial venous carbon dioxide pressure; SID_6_ Measured strong ion difference measured using 6 electrolytes; USI, unmeasured strong ions.

### 3.4 Factors associated with mortality in hospitalized sick goats

One‐hundred sixteen (81%) goats survived to hospital discharge whereas 27 (19%) did not. Six (22%) of the 27 nonsurviving goats died during hospitalization and 21 (80%) were euthanized. Eight goats were euthanized (n = 7) or died (n = 1) on day 1 of hospitalization. The goat that died on day 1 of hospitalization was being treated for haemonchosis by blood transfusion. Diagnoses of euthanized goats on day 1 of hospitalization included end stage liver disease (n = 2), severe trauma from dog attack (n = 1), urolithiasis (n = 1), diarrhea (n = 1), and chronic bronchopneumonia (n = 1). Diagnoses of the goats that died during hospitalization (n = 6) included haemonchosis (n = 2), peritonitis (n = 1), meningitis (n = 1), pregnancy toxemia (n = 1), and bronchopneumonia (n = 1). Diagnoses of the goats that were euthanized during hospitalization (n = 20) included neoplasia (n = 3), end stage liver disease (n = 2), emaciation (n = 2) pregnancy toxemia (n = 2), urolithiasis (n = 2), diarrhea (n = 1), nephrotic syndrome (n = 1), severe trauma from dog attack (n = 1), caprine arthritis‐encephalitis (n = 1), vagal indigestion (n = 1), toxic mastitis (n = 1), chronic pneumonia (n = 1), listeriosis (n = 1), and dystocia (n = 1). The time of hospitalization (median and range) for surviving goats was 3 days (0‐20 days) and for nonsurviving goats was 2 days (0‐9 days; *P* = .17).

Admission values (median and range) of blood gas, electrolytes, acid‐base and selected laboratory variables of surviving and nonsurviving sick goats are presented in Supplementary Table 2. Variables associated with mortality in the univariable analysis were HR <80 bpm, RR <16 rpm, HCO_3_^−^ <20 mmol/L, glucose, blood pH, SID_6_, and USI. Blood pH and USI were highly correlated (Supplementary Table 3) and therefore 2 different multivariable Cox proportional hazard models were built using each of those variables (Table 5). The first multivariable Cox proportional model assessing the association between selected variables and survival included RR <16 rpm and USI (Table 5). The model correctly ordered survival times for pairs of goats 68% of the time (Harrell's *C* concordance statistic, 0.68). The optimal cut point for USI was determined to be −0.49 mmol/L based on Youden's index. At this cut point, the sensitivity, specificity and area under the ROC curve for predicting nonsurvival was 52%, 69%, and 0.60, respectively. The second multivariable Cox proportional model using blood pH for predicting hospital mortality included the variables RR <16 rpm and blood pH. The SID_6_ was retained in the latter model because it changed the coefficient of pH by >20% indicating a potential confounding effect (Table 5). The Harrell's *C* concordance statistic was 0.66, indicating the model could correctly order survival times for pairs of goats 66% of the time based on pH, RR, and SID_6_. The optimal cut point for pH was determined to be 7.45 mmol/L. At this cut point, the sensitivity, specificity, and area under the ROC curve for predicting nonsurvival was 56%, 53%, and 0.55, respectively.

**TABLE 5:** Results of the multivariable Cox proportional hazard models assessing the association between selected admission clinical and laboratory variables and outcome of 143 sick goats

Variable		HzR	SE	95% CI	*P* value
Model 1					
USI (mEq/L)	Every 1 mEq/L USI unit increase	1.13	0.05	1.04‐1.22	.004
RR (rpm)	Normal (16‐30)	Referent			
	>30	1.14	0.55	0.45‐2.93	.78
	<16	9.62	8.33	1.76‐52	.009
Model 2					
RR (rpm)	Normal (16‐30)	Referent			
	>30	1.09	0.52	0.43‐2.8	.86
	<16	7.50	6.47	1.38‐41	.02
Blood pH	Every 0.01 pH unit increase	0.93	0.03	0.89‐0.98	.01
SID_6_ (mEq/L)	Every 1 mEq/L SID_6_ unit increase	1.07	0.04	0.99‐1.16	.08

Abbreviations: CI, confidence interval; HzR, hazard ratio; RR, respiratory rate; SID, strong ion difference.

## 4 DISCUSSION

We found that hypocapnia, hypokalemia, hyperchloremia, hyperlactatemia, and hyperproteinemia were common abnormalities identified in sick goats, and that respiratory alkalosis, SID_6_ acidosis, and A_tot_ acidosis were acid‐base disorders frequently encountered in hospitalized goats. The results also indicated that changes in RR (<16 rpm), USI, and blood pH on admission were associated with hospital mortality in sick goats. According to our results, *s*SID variables accounted for most of the changes in blood pH and HCO_3_^−^. The *s*SID variables explained 97% and 100% of the changes in blood pH and HCO_3_^−^, respectively. Similar results were reported in sick calves^9^, ^10^ and foals.^5^ In calves, with or without diarrhea, *s*SID contributed to 85% to 93% of the change in blood pH and 98% of the change in HCO_3_^−^, whereas in foals, *s*SID variables explained 83% to 94% of the changes in blood pH.

Our findings are in agreement with previous research in cattle and horses showing that the *s*SID approach provides an accurate evaluation of acid‐base homeostasis because it was able to detect complex mixed alteration in SID_6_, P_v_CO_2_, USI, and A_tot_.^27^ The H‐H approach states that primary respiratory and metabolic disturbances compensate for each other. For example, a respiratory disorder will be compensated by renal excretion or reabsorption of HCO_3_, whereas a metabolic disorder will be rapidly compensated by alteration in pCO_2_ by the respiratory system. Describing primary and compensatory responses to acid‐base disturbances using HCO_3_^−^ is qualitative and offers little information of the physiological status of the patient.^30^ The *s*SID approach determines quantitatively how a primary acid‐base disturbance in PCO_2_, A_tot_ or SID_6_ produces an effect on the other 2 variables to initiate a compensatory response. The *s*SID approach states that SID_m_ (SID_6_ in our study) has a normal value of approximately 40 mmol/L in a single compartment system where A_tot_ and PCO_2_ remain constant at normal values.^12^, ^13^, ^14^ However, a codependency between SID_m_, PCO_2_, and A_tot_ exists when the system is defined as whole body acid‐base balance.^30^ Thus, the respiratory alkalosis (hypocapnia) observed in the majority of the goats in our study could be a primary physiological (eg, pregnancy, pain, and stress) or a pathological (eg, pneumonia, sepsis, and anemia) alteration, resulting in increased alveolar ventilation. Hypocapnia as a primary alteration leads to changes in all body H^+^ activity including that in plasma, intracellular and interstitial fluids. An acute, but especially chronic, change in PCO_2_ stimulates alterations in SID_m_ that are adjusted mostly by alteration in the Na^+^ and Cl^−^ ratio by kidney regulation of Cl^−^.^13^, ^31^, ^32^, ^33^, ^34^ It is likely that during respiratory alkalosis in sick goats, the renal excretion of Cl^−^ decreases, resulting in hyperchloremia, and subsequently lowering SID_m_. Therefore, in cases of primary respiratory alkalosis, SID_6_ acidosis should not be interpret as a mixed acid‐base disorder, but rather as a new “normal” SID_6_ value.^30^

In a subset of goats enrolled in our study, respiratory alkalosis could be a compensatory response to the changes in blood pH because of SID_6_ acidosis, USI acidosis, or A_tot_ acidosis. Changes in SID_6_ could result from a high [Cl^−^] relative to [Na^+^] (eg, acute diarrhea, mastitis), increased [L‐lac^−^] (eg, haemonchosis, mastitis, and dystocia) or increased USI (eg, BHB in pregnancy toxemia). The effect of high [Cl^−^] relative to [Na^+^] on blood pH and HCO_3_^−^ has been demonstrated in children,^34^ calves,^9^, ^10^, ^11^ horses with diarrhea,^6^ critically ill calves^9^ and foals with sepsis.^5^ In fact, in diarrheic horses, fecal fluid [Cl^−^] losses were lower than those of [Na^+^] and urinary excretion of [Cl^−^] was decreased.^35^ Plasma [Cl^−^] then increases, lowering SID_m_, and causing SID acidosis.^36^ The reasons for the hyperchloremia in goats with mastitis and pneumonia are not clear, but those goats usually presented to our institution with signs of sepsis. Similarly, in humans with severe sepsis and septic shock, hyperchloremic metabolic acidosis is diagnosed frequently.^37^

Hyperlactatemia was diagnosed commonly in sick goats, especially diarrheic goats and those with haemonchosis. Several studies in humans,^38^, ^39^ cattle,^12^, ^40^, ^41^, ^42^ horses,^6^, ^43^ and dogs^44^ have reported hyperlactatemia to be present in the majority of critically ill patients. L‐lactate is a strong anion and its increased plasma concentration lowers the SID_m_, leading to SID_6_ acidosis. Hyperlactatemia traditionally has been attributed to dehydration and tissue hypoxia.^45^ Therefore, hypoperfusion caused by dehydration could have been responsible for the hyperlactatemia in the goats. However, several studies have challenged this hypothesis because hyperlactatemia can occur in presence of normal tissue oxygenation. It has been proposed that hyperlactatemia in critically ill patients is associated with enhanced aerobic glycolytic flux, which is the most important determinant of lactate production.^46^ Increased aerobic glycolytic flux in humans with sepsis and septic shock is enhanced by epinephrine secretion^47^, ^48^, ^49^ and respiratory alkalosis.^50^ Additionally, in macrophage‐rich organs including the lungs, intestine, and liver (ie, pneumonia, diarrhea, and hepatitis, respectively), inflammatory cytokines (ie, tumoral necrosis factor and interleukin 1) promote aerobic glycolytic flux for adenosine triphosphate production required for respiratory burst during sepsis.^51^ Thiamine (vitamin B_1_) deficiency negatively impacts oxidative energy production^49^ and enhances aerobic glycolytic flux.^50^ In ruminants on well‐balanced diets, thiamine is produced by bacteria in the rumen but during periods of anorexia or hyporexia, thiamine deficiency can occur. Thus, it can be hypothesized that in the subset of sick goats included in our study, thiamine deficiency could have contributed, at least in part, to tissue hyperlactatemia.^52^, ^53^ Propylene glycol (PG) commonly is administered to sick goats, especially those suffering from pregnancy toxemia. It is metabolized to lactate and excreted by the liver or kidneys. Therefore, administration of PG to goats with hepatic or renal insufficiency also could have contributed to the hyperlactatemia.^54^

In our study, changes in TPP also were associated with acid‐base disorders in which hypoproteinemia and hyperproteinemia lead to A_tot_ alkalosis and acidosis, respectively.^12^, ^13^ The A_tot_ alkalosis in goats with haemonchosis likely resulted from protein‐losing enteropathy, whereas it could have resulted from volume expansion (which occurs during pregnancy) in goats with dystocia.^55^ In sick goats, A_tot_ acidosis was diagnosed frequently, likely because of a decrease in plasma water content because of decreased water intake and loss of protein‐poor body fluids (eg, hypersecretory diarrhea). Of the goats that presented to our institution with mastitis, most were acute cases, but in chronic cases it is possible that hyperglobulinemia could have affected the TPP concentration, leading to A_tot_ acidosis.

Contrary to the H‐H approach, the *s*SID approach offers a calculation to determine the USI and their effect on blood pH and HCO_3_. In our study, USI acidosis was detected in the majority of goats with pregnancy toxemia likely because of increased concentration of BHB. Four goats included in the “other” diseases group also had USI acidosis, 1 goat had end‐stage liver disease, 2 had polytraumatic lesions associated with dog attack, and 1 had peritonitis. The source and nature of the USI in critically ill patients is unknown, but their concentrations are increased in humans and calves with liver and kidney injuries ^17^, ^20^ and in people with severe trauma.^56^ Proposed sources of USI in critically patients include intermediate products from the Kreb's cycle^57^, ^58^ and exogenous anionic compounds in antimicrobials or electrolytic solutions.^59^ Changes in plasma phosphate and protein concentrations affect A_tot_ calculation and, therefore, the USI concentration.^9^, ^12^ Marked increases in plasma phosphate concentration^11^ and production of acute phase inflammatory proteins^17^ could contribute, at least in part, to the increase in USI. Finally, USI also could include intracellular anions that are released into the systemic circulation during endotoxemia.

The concentration of USI and values of blood pH on admission were associated with higher hazard ratios of hospital mortality in sick goats. Our models indicated that for every 1 mEq/L increase in USI, measured at admission, the hazard for nonsurvival, at any point in time during hospitalization, increased by 13%. In addition, for each 0.01 pH unit increase measured at admission, the hazard for nonsurvival to hospital discharge decreased by 6.6%. The experimental design of our study prevented us from identifying a true cause and effect relationship between pH and USI and mortality, but acidemia and USI acidosis are important markers of disease severity in human^15^, ^19^, ^56^, ^57^, ^58^, ^59^, ^60^, ^61^ and veterinary^7^, ^25^, ^62^, ^63^ critically ill patients. Of interest, USI was a better predictor for hospital mortality of humans with blunt and penetrating trauma than were pH, HCO_3_^−^, L‐lac^−^, AG corrected by albumin and standard base excess.^60^ Thus, clinicians should consider measured pH and calculated USI in sick goats, but the predictive accuracy of assessing these metrics alone is low with low sensitivity and specificity in identifying non‐survivors. Therefore, it is necessary to assess both USI and pH in light of the battery of information required to establish a treatment plan and a patient's prognosis.

Our study investigated the acid‐base status of a selected group of sick goats using the sSID approach, and the clinicopathological variables associated with the mortality of sick goats. The results likely were biased toward sicker patients because the sample included only sick goats referred to a single teaching hospital. Therefore, our results cannot be directly extrapolated to any other population. However, the cases admitted to our teaching hospital included sick goats with a variety of diseases similar to those admitted to other tertiary referral hospitals. The retrospective design of our study prevented identification of preanalytical effects such as the exact time from sample collection to processing,^64^ the type^64^ and size^65^ of evacuated blood collection tube, and the potential for underfilling of the tube^65^ on the measured variables, especially P_v_CO_2_, P_v_O_2_, and total CO_2_ concentration. Generally, at our institution blood sample collection is performed using 2 mL plastic tubes containing sodium heparin. The collection is continued until blood stops flowing and aspiration of air into the tube is avoided during the process. The tubes are inverted several times to mix the blood with the anticoagulant, and the collected samples are processed immediately. Although this sampling process could have minimized the effects of preanalytical factors on the measured variables, the results of our study should be interpreted taking this limitation into consideration.

Because of the retrospective nature of our study, data collection was not standardized, and categorization of clinical signs and treatments was not possible. This prevented assessment of the effect of individual treatment variations on the survival analysis. The outcome of the survival analysis could have been affected by the number of euthanized goats included in the nonsurvival group. However, the diagnosis of most euthanized goats carried a poor prognosis even in the presence of unlimited economic resources. A recent study^63^ investigating factors predicting mortality in 1400 sick calves with different diseases and diarrhea conducted an analysis of nonsurvival calves by allocating them to the survival group if unlimited financial resources would likely have resulted in their survival. The results of this analysis showed that approximately 80% of the euthanized calves were likely to have died despite the availability of unlimited financial resources. This observation suggests that, in the majority of cases, the clinician's recommendation of patient euthanasia is appropriate and appears to be based on the probability of the animal's survival and welfare rather than economic constraints.

In conclusion, we showed that the *s*SID approach is a useful methodology to quantify acid‐base disorders in goats and to determine their mechanism of development. We also demonstrated that USI and pH can aid in establishing the prognosis of sick goats. Additional investigations evaluating the dynamic of USI during hospitalization and their association with morbidity and mortality in sick goats are warranted.

## CONFLICT OF INTEREST DECLARATION

Authors declare no conflict of interest.

## OFF‐LABEL ANTIMICROBIAL DECLARATION

Authors declare no off‐label use of antimicrobials.

## INSTITUTIONAL ANIMAL CARE AND USE COMMITTEE (IACUC) OR OTHER APPROVAL DECLARATION

Authors declare no IACUC or other approval was needed.

## HUMAN ETHICS APPROVAL DECLARATION

Authors declare human ethics approval was not needed for this study.

## Supporting information


**Supplementary Table 1** Admission values of selected clinical and laboratory variables of 143 sick goats.

**Supplementary Table 2** Admission values (median and range) of blood gas, electrolytes, acid‐base and selected laboratory variables of 143 sick goats.

**Supplementary Table 3** Results of the univariable Cox proportional hazard models assessing the association between selected admission clinical and laboratory variables and outcome of 143 sick goats.
