# Supplementary material for: Acid‐base disorders in sick goats and their association with mortality: A simplified strong ion difference approach
Source: J Vet Intern Med. 2020 Nov 3;34(6):2776–86. doi: 10.1111/jvim.15956 (PMC7694813; doi:10.1111/jvim.15956)
Supplement: Supplementary file 1 — Supplementary Table 1 Admission values of selected clinical and laboratory variables of 143 sick goats. [file JVIM-34-2776-s001.pdf]

Supplementary Table 1. Admission values of selected clinical and laboratory variables of 143 sick goats.

Variable	N	Mean	SD	Median	Minimum	Maximum
Age (years)	140	3.0	2.7	2.0	0.25	14
Respiration (rpm)	137	52	30	40	10	120
Pulse (bpm)	140	123	30	124	44	184
Temperature (°F)	138	102	2	102	93	106
PCV (%)	143	28	10	29	7.0	59
TPP (g/dL)	143	6.4	1.1	6.5	2.4	9.4
Glucose (mmol/L)	142	131	72	115	23	389
Globulin (g/dL)	44	4.3	0.95	4.3	2.9	7.0
Albumin (g/dL)	44	2.4	0.73	2.5	0.5	3.4
BUN (mmol/L)	44	29	25	21	4.0	158
Creatinine (g/dL)	140	1.7	2.3	1.0	0.4	17
Na^+ (mmol/L)	143	142	4.3	143	126	152
K^+ (mmol/L)	143	3.9	1	3.9	2.0	10
Cl^- (mmol/L)	143	110	5.0	111	87	120
Ca^{2+} (mmol/L)	143	1.1	0.13	1.1	0.8	1.6
Mg^{2+} (mmol/L)	143	0.5	0.13	0.5	0.3	1.3
Phosphorus (mmol/L)	44	6.0	4.3	5.5	1	27
L-lactate$^-$ (mmol/L)	143	2.7	2.2	2.2	0.2	13
pH	143	7.44	0.06	7.45	7.18	7.57
$pvCO_2$ (mmHg)	143	28	5.3	28	16	59
HCO_3^- (mmol/L)	143	20	4.4	20	7.7	35
SID_6 (mmol/L)	143	34	4.8	34	18	50
A_{tot} (mmol/L)	143	22	4	22	8.2	32
USI (mmol/L)	143	-0.75	3.2	-1.3	-5.1	14
SIG (mmol/L)	143	-0.31	3.9	0.6	-17.1	6.7
AG (mmol/L)	143	15	3.8	15	5.3	35

SD, standard deviation; rpm, respirations per minute; bpm, beats per minute; °F, fahrenheit degrees; PCV, packet cell volume; TP, total plasma proteins; HCO_3^-, bicarbonate; $pvCO_2$, venous partial carbon dioxide pressure; AG, anion gap; SID, strong ion difference; SIG, strong ion gap; USI, unmeasured strong ions; A_{tot}, total plasma concentration of nonvolatile weak acids.
