# Supplementary material for: Acid‐base disorders in sick goats and their association with mortality: A simplified strong ion difference approach
Source: J Vet Intern Med. 2020 Nov 3;34(6):2776–86. doi: 10.1111/jvim.15956 (PMC7694813; doi:10.1111/jvim.15956)
Supplement: Supplementary file 2 — Supplementary Table 2 Admission values (median and range) of blood gas, electrolytes, acid‐base and selected laboratory variables of 143 sick goats. [file JVIM-34-2776-s002.pdf]

Supplementary Table 2. Admission values (median and range) of blood gas, electrolytes, acid-base and selected laboratory variables of 143 sick goats.

**Variable**	**Survivors n = 116**	**Non-survivors n = 27**
Age (years)	2 [0.25 to 14]	3 [0.35 to 10]
Temperature (°F)	102 [93 to 106]	102 [95 to 105]
Pulse (bpm)	128 [52 to 184]	120 [44 to 180]
Respiration (rpm)	40 [12 to 120]	36 [10 to 120]
PCV (%)	28 [7 to 60]	29 [8 to 47]
TP (g/dL)	6.5 [3.5 to 9.4]	6.5 [2.4 to 8.4
Glucose (mg/dL)	118 [23 to 389]	91 [30 to 301]
Creatinine (mg/dL)	0.9 [0.4 to 17]	1 [0.4 to 6]
Na^+ (mmol/L)	143 [126 to 152]	143 [130 to 152]
K^+ (mmol/L)	3.9 [2 to 10]	3.5 [2.3 to 5.5]
Cl^- (mmol/L)	111 [87 to 120]	110 [100 to 118]
Ca^{2+} (mmol/L)	1.1 [0.8 to 1.6]	1.1 [0.8 to 1.5]
Mg^{2+} (mmol/L)	0.49 [0.3 to 1.3]	0.5 [0.3 to 0.9]
L-lactate$^-$ (mmol/L)	2.2 [2 to 12]	1.7 [0.3 to 8.3]
pH	7.453 [7.18 to 7.57]	7.457 [7.22 to 7.54]
$PvCO_2$ (mmHg)	28 [16 to 59]	27 [18 to 46]
HCO_3^- (mmol/L)	20 [8 to 33]	20 [8 to 35]
AG (mEq/L)	15 [6.5 to 25]	15 [5.3 to 35]
SID_6 (mEq/L)	34 [19 to 49]	35 [26 to 50]
A_{tot} (mmol/L)	22 [12 to 32]	22 [8 to 29]
USI (mEq/L)	-1.4 [-5.1 to 6.6]	-0.4 [-5 to 14]

Data presented as median and range. PCV, packet cell volume; TP, total plasma proteins; HCO_3, bicarbonate; $PvCO_2$, venous partial carbon dioxide pressure; AG, anion gap; SID, strong ion difference; SIG, strong ion gap; USI, unmeasured strong ions; A_{tot}, total plasma concentration of nonvolatile weak acids.
