# Supplementary material for: Acid‐base disorders in sick goats and their association with mortality: A simplified strong ion difference approach
Source: J Vet Intern Med. 2020 Nov 3;34(6):2776–86. doi: 10.1111/jvim.15956 (PMC7694813; doi:10.1111/jvim.15956)
Supplement: Supplementary file 3 — Supplementary Table 3 Results of the univariable Cox proportional hazard models assessing the association between selected admission clinical and laboratory variables and outcome of 143 sick goats. [file JVIM-34-2776-s003.pdf]

**Supplementary Table 3. Results of the univariable cox proportional hazard models assessing the association between selected admission clinical and laboratory variables and outcome of 143 sick goats.**						
**Variable**		**Hazard Ratio**	**Std. Err.**	**P - value**	**95% Confidence interval**	
					**Lower**	**Upper**
Sex	Female	*Referent*				
	Male	0.7182	0.5388	0.659	0.1650	3.1253
	Wether	1.7572	0.7899	0.21	0.7280	4.2410
Temperature (°F)	101.3 to 103.5	*Referent*				
	< 101.3	1.0152	0.4973	0.975	0.3886	2.6516
	> 103.5	1.2939	0.6372	0.601	0.4928	3.3970
Heart rate (bpm)	70 to 80	*Referent*				
	< 70	0.9678	0.7178	0.965	0.2261	4.1416
	> 80	3.5820	3.2925	0.165	0.5912	21.703
Respiratory rate (rpm)	16 to 30	*Referent*				
	< 16	6.7544	5.6649	0.023	1.3052	34.953
	> 30	0.9971	0.4703	0.995	0.3956	2.5132
K^+ (mmol/L)	4.2 to 6	*Referent*				
	< 4.2	8.9^{e-01}	3.6^{e-01}	0.783	0.3965	2.0074
Cl^- (mmol/L)	98 to 110	*Referent*				
	< 98	0.5938	0.2369	0.20	0.2725	1.2941
	> 110					
Creatinine (mg/dL)	< 0.8	*Referent*				
	> 0.8	2.0744	1.0347	0.144	0.7803	5.5142
HCO_3 (mmol/L)	20 to 30	*Referent*				
	< 20	3.4508	2.2829	0.061	0.9436	12.619
	>30	1.2724	0.5361	0.567	0.5571	2.9060
Anion Gap (mEq/L)	< 24	*Referent*				
	>24	1.9618	1.4671	0.368	0.4529	8.4960
pH	-	0.0088	0.0235	0.076	0.0000	1.6357
PCO_2 (mmHg)	-	1.0397	0.0330	0.221	0.9768	1.1066
Packet cell volume (%)	-	1.0037	0.0197	0.850	0.9657	1.0431
Total solids (mg/dL)	-	0.8657	0.1477	0.398	0.6196	1.2097
Hemoglobin (mg/dL)	-	1.1190	0.1159	0.278	0.9133	1.3710
Ca^{2+} (mmol/L)	-	1.2223	1.8054	0.892	0.0676	22.100
Mg^{2+} (mmol/L)	-	0.7426	1.3123	0.866	0.0232	23.710
Glucose (mg/dL)	-	0.9952	0.0034	0.176	0.9884	1.0021
L-Lactate (mmol/L)	-	1.0648	0.0914	0.464	0.8999	1.2600
SID_6 (mEq/L)	-	1.0581	0.0447	0.182	0.9739	1.1496
USI (mEq/L)	-	1.1141	0.0458	0.009	1.0278	1.2076
